# Recent advances in pulmonary arterial hypertension

**DOI:** 10.12688/f1000research.14984.1

**Published:** 2018-07-24

**Authors:** Martin R. Wilkins, Jurjan Aman, Lars Harbaum, Anna Ulrich, John Wharton, Christopher J. Rhodes

**Affiliations:** 1 Department of Medicine, Imperial College London, London, UK

**Keywords:** pulmonary hypertension, BMPR2, bone morphogenetic receptor type 2, new drug targets

## Abstract

Pulmonary arterial hypertension (PAH) is a rare disorder with a high mortality rate. Treatment options have improved in the last 20 years, but patients still die prematurely of right heart failure. Though rare, it is heterogeneous at the genetic and molecular level, and understanding and exploiting this is key to the development of more effective treatments. *BMPR2*, encoding bone morphogenetic receptor type 2, is the most commonly affected gene in both familial and non-familial PAH, but rare mutations have been identified in other genes. Transcriptomic, proteomic, and metabolomic studies looking for endophenotypes are under way. There is no shortage of candidate new drug targets for PAH, but the selection and prioritisation of these are challenges for the research community.

## Introduction

The normal adult pulmonary circulation, in contrast to the systemic circulation, is a low-pressure, low-resistance vascular bed. Pulmonary hypertension (PH) is diagnosed when the resting mean pulmonary artery pressure (mPAP) is at least 25 mmHg and is classified into five main subgroups based on clinical and haemodynamic criteria ^1^. It leads to an increased workload for the right ventricle, which, if it fails to hypertrophy and adapt, can result in premature death.

**Figure 1.**
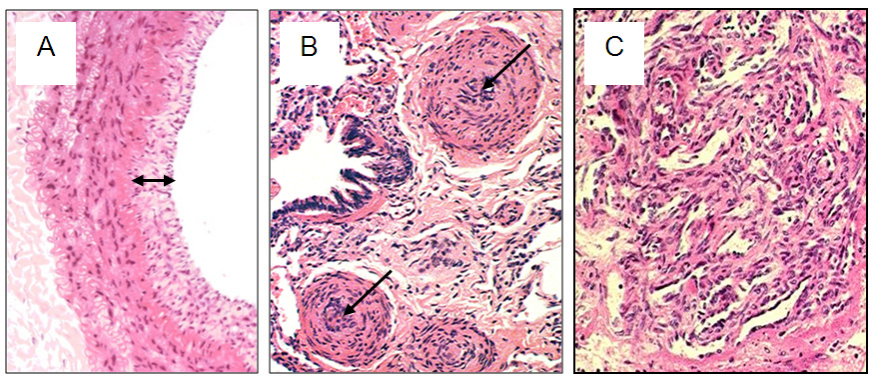
Vascular remodelling in pulmonary arterial hypertension. Haematoxylin-and-eosin staining showing ( **A**) neointimal proliferation (double arrow) in an elastic pulmonary artery, ( **B**) medial hypertrophy and neointimal proliferation leading to occlusion of the vessel lumen (arrows) in muscular pulmonary arteries, and ( **C**) a plexiform lesion, comprising a plexus of capillary-like channels, in a patient with plexogenic arteriopathy.

PH is not uncommon in left heart failure, in which there is an increasing appreciation of a gradation of risk such that even borderline elevation of mPAP contributes to mortality ^2^. Pulmonary arterial hypertension (PAH) occurs less frequently—it has a reported incidence of 1.1 to 17.6 per million adults per year and a prevalence of 6.6 to 26.0 per million adults ^1^ —and is diagnosed when the elevated mPAP is attributed to pre-capillary resistance to pulmonary blood flow, in the absence of airway or parenchymal lung diseases, or chronic thromboembolism ^1, 3^. PAH is clinically heterogeneous, comprising patients presenting with PAH with no obvious cause (termed as idiopathic PAH [IPAH]), heritable PAH, drug-induced PAH, and PAH with associated congenital heart disease, connective tissue disease, HIV, portal hypertension or schistosomiasis. Histology at post-mortem or lung transplantation reveals marked pulmonary arterial remodelling due to vascular cell proliferation encroaching on the vascular lumen ( Figure 1).

Even in the modern era, with licensed medication in four drug classes, annual mortality from PAH remains high, at about 10% per year ^1^. The past two decades have seen a concentrated effort from academia and industry to better understand PAH and improve treatment. The limitations of animal models ^4^ have emphasised the importance of patient-orientated studies, and international collaboration is required to bring together well-phenotyped cohorts of patients. Here, we summarise the progress that is being made from these efforts.

## Genetics

Genetic studies have focused on IPAH, heritable PAH, and drug-induced PAH. Pathogenic variants in *BMPR2* (encoding bone morphogenetic receptor 2), first reported in 2000 ^5, 6^, are found in up to 80% of patients with a clinical diagnosis and a family history of PAH and 15% of patients with IPAH and no known family history ^7^. Mutations in *BMPR2* increase susceptibility to PAH (lifetime penetrance is 20%) and are associated with an earlier age of onset of PH and a worse prognosis ^8^. In the past 18 years, family studies, isolated cases, small case series, and a recent genome-wide case-control analysis of patients in the National Institute for Health Research BioResource (NIHRBR) Rare Diseases study ^7^ have linked at least 10 other genes to PAH ( Table 1). Some, such as *ACVRL1*, *ENG*, *SMAD9*, and *GDF2*, encode proteins in the transforming growth factor-beta (TGFβ) superfamily and emphasise the significance of this signalling pathway in pulmonary vascular homeostasis. Others, such as *KCNK3* ^9^, *TBX4* ^10^, and newly identified genes *ATP13A3*, *AQP1*, and *SOX17* ^7^, point to genetic heterogeneity in PAH and novel pathways for therapeutic intervention.

**Table 1.** Genes containing rare variants associated with pulmonary arterial hypertension.

Code	Name
*BMPR2*	Bone morphogenetic protein receptor type 2
*ACVRL1*	Activin A receptor like type 1
*ENG*	Endoglin
*SMAD9*	SMAD family member 9
*KCNK3*	Potassium two pore domain channel subfamily K member 3
*TBX4*	T-box 4
*GDF2*	Growth differentiation factor 2 (or bone morphogenetic protein 9)
*ATP13A3*	ATPase 13A3
*AQP1*	Aquaporin 1
*SOX17*	SRY-box 17
*EIF2AK4* ^a^	Eukaryotic translation initiation factor 2 alpha kinase 4

Rare causal variants identified by family studies and whole genome sequencing case-control analyses ^6^. ^a^ *EIF2AK4* mutations associated with pulmonary veno-occlusive disease and pulmonary capillary haemangiomatosis ^10^.

The UK NIHRBR study, which has sequenced over 14,000 whole genomes, including 1,048 cases with a clinical diagnosis of IPAH, heritable PAH, or drug-associated PAH and 6,385 non-PAH controls of European ancestry ^7^, has offered powerful insight into the genetic architecture of clinical PAH. An early observation is that 1% of patients diagnosed with IPAH in the UK cohort have biallelic mutations in *EIF2AK4*, a gene associated with pulmonary veno-occlusive disease (PVOD) ^11^. As patients with PVOD have a poorer prognosis and treatment response, this example illustrates the relevance of genetics for accurate subclassification of patients with PAH, and genetic testing for *EIF2AK4* should be considered in patients presenting with IPAH. Further studies of larger cohorts will be needed to identify other rare causative mutations. Given the rarity of the disorder, international collaboration will be essential to determine the contribution of common genetic variation to disease risk and outcomes in PAH.

## Molecular signatures of pulmonary arterial hypertension

It is accepted that the current clinical classification of PAH is unsatisfactory, from the point of view of both diagnosis and developing new drugs. Genetics coupled with deep molecular phenotyping offers the hope of defining the key drivers of PAH and new drug targets, opening up the possibility of more personalised medicine. The associated National PAH Cohort Study ( http://www.ipahcohort.com) is facilitating the collection of biological samples from PAH patients across the UK and is beginning to provide deep phenotyping data, such as metabolomics and proteomics, which can be cross-referenced with the genetic data provided by the NIHRBR effort ^12, 13^.

A subgroup of patients with PAH, less than 10%, respond well to calcium channel blockers, suggesting that this pharmacological phenotype should have a distinct molecular signature. In support of this, a transcriptomic signature has been reported in a small group of patients which requires further prospective validation ^14^. Responders to calcium channel blockers have also been demonstrated to have metabolic profiles more similar to those of healthy volunteers than PAH patients who do not respond ^13^.

High-throughput technologies, such as that provided by aptamer-based assays, nuclear magnetic resonance, and mass spectroscopy, have been applied to plasma samples and used to risk-stratify patients ^12, 13^. The prognostic panels developed from this approach may add valuable information to clinical assessment. For example, a nine-protein panel was shown to improve risk stratification in combination with either N-terminal pro-brain natriuretic peptide (NT-proBNP) or the REVEAL (Registry to EValuate Early And Long-term PAH) registry risk equation, an equation built from clinical assessments and comorbidities of patients ^12, 15^. The advantage of a panel of circulating biomarkers is that they are more objective than functional class assessment and more accessible than imaging, and the combination of molecules reporting on different pathologies (for example, proliferation, inflammation, coagulation, and metabolic dysfunction) provides greater detail than a single biomarker (for example, brain natriuretic peptide).

But the real power of these techniques lies in their potential for identifying important and therapeutically relevant subgroups of patients presenting in the clinic. A US National Heart, Lung, and Blood Institute-funded initiative is looking to explore this. The PVDOMICS (Pulmonary Vascular Disease Phenomics Program) consortium seeks to “redefine pulmonary hypertension through pulmonary vascular disease phenomics” ^16^. The aim is to enrol 1,500 participants with PH and healthy comparators for comprehensive clinical and “omic” analyses. Recruitment has begun and an analysis plan has been outlined (ClinicalTrials.gov Identifier: NCT02980887). The challenge of data integration is not to be underestimated, but if successful it will provide the basis for a molecular classification of PH and biologically important insights.

## New drugs

The current treatments for PAH come from four drug classes (prostanoid analogues, endothelin receptor antagonists, phosphodiesterase type 5 inhibitors, and soluble guanylate cyclase stimulators) that act to address endothelial dysfunction and reduce vasomotor tone. They provide symptom relief and improve functional capacity, but there is limited evidence that these drugs arrest the course of PAH and prolong the survival of patients. To do that, a drug will need to at least support right ventricular function and, better still, reverse the remodelling of pulmonary arteries. There is no shortage of potential new drug targets for PAH.

### Targets from genetics

Impaired BMPR2 signalling creates an imbalance in TGFβ/BMP signalling favouring TGFβ and may underlie vascular remodelling in PAH patients with and without *BMPR2* mutations. A number of therapeutic strategies have been proposed, beyond the aspiration of gene therapy, and include pharmacological approaches, such as chloroquine (to prevent lysosomal degradation of the BMPR2) ^17^, ataluren (to read through missense mutations), and increasing BMP9 levels ^18^. To date, the only treatment that has been used to target *BMPR2* signalling in clinical trials is tacrolimus. This drug binds and removes FKBP12 from all three BMP type 1 receptors and activates BMPR2-mediated signalling even in the absence of exogenous ligand and *BMPR2*. Although some patients responded with a pronounced increase in *BMPR2* expression as well as improvement in 6-minute walk distance (6MWD) and serological and echocardiographic parameters of heart failure, the changes were not observed across all patients ^19^. An alternative approach is to inhibit TGFβ activity by using a novel activin-receptor fusion protein (sotatercept) that competitively binds and neutralises TGFβ-superfamily ligands ^20^. This approach is progressing to clinical trials, but the effect of the rise in haematocrit that accompanies this therapy needs to be carefully monitored and understood.

### Growth factors

Much has been made of the similarities between the dysregulated growth of vascular cells and that of tumour cells, leading to interest in repurposing drugs from oncology. Studies with the tyrosine kinase receptor inhibitor imatinib have led the field. In addition to inhibiting the bcr-abl tyrosine kinase, imatinib inhibits platelet-derived growth factor (PDGF) receptor-α and -β and c-KIT. PDGF is a trophic factor in vascular cells, and PAH lung shows increased expression of PDGF receptors ^21^. A phase 3 trial reported an increase in mean placebo-corrected treatment effect on 6MWD of 32 m and a reduction in pulmonary vascular resistance by 379 dyne·s·cm ^−5^ ^22^. But there was no improvement in time to clinical worsening, and serious adverse events and discontinuations were more frequent with imatinib. Of particular concern was subdural hematoma, which occurred in eight patients receiving imatinib and anticoagulation. Further development of imatinib as a treatment has halted, but there remains interest in understanding the characteristics of patients who appear to derive considerable benefit. The ability to identify potential responders, coupled with avoiding concomitant anticoagulation, would help the argument to revisit imatinib as a treatment. However, there remains concern over toxicity, as other tyrosine kinase inhibitors have fared less well and dasatinib use for other indications is associated with the development of PH on rare occasions ^23, 24^.

There is an active interest in elastase inhibitors, which have been shown to prevent and reverse experimental PH ^25, 26^. Elafin, an endogenously produced low-molecular-weight elastase inhibitor, induces apoptosis in human pulmonary arterial smooth muscle cells and decreases neointimal lesions in lung organ culture. Augmentation of BMPR2 signalling, dependent upon stabilisation of caveolin-1 in the cell membrane, has also been implicated as a mechanism of action ^26^.

### Metabolism

The “metabolic theory” of PAH is based on the observation that proliferating cells switch cell metabolism from oxidative phosphorylation to glycolysis for ATP production ^27^. Increased expression of pyruvate dehydrogenase kinase (PDK), which inhibits pyruvate dehydrogenase, is one factor underlying this switch, but other PDK-independent factors (for example, variants reducing the function of SERT3 or UCP2 that predict reduced protein function) may also contribute ^28, 29^ and indeed impair the clinical response to inhibition of PDK by dichloroacetate (DCA) ^30^. A 16-week study of DCA treatment in PAH showed a reduction in pulmonary artery pressure alongside a reduction in lung parenchymal glucose uptake in genetically susceptible patients ^30^.

Aside from the metabolic perturbation seen in proliferating cells, insulin resistance is common in PAH ^31^. Although the underlying explanation for this association is not clear, pre-clinical data support further evaluation of repurposing insulin resistance medicines in PAH. These include rosiglitazone ^32^, metformin ^33^, and glucagon-like peptide 1 (GLP-1) receptor agonists ^34^.

### Oestrogen signalling

Despite disparities in the results of pre-clinical and clinical studies, modulation of oestrogen levels is another potential therapeutic approach for the treatment of PAH ^35^. A variant in the promoter region of the aromatase gene, which encodes the enzyme responsible for the conversion of androgens to oestrogen, is associated with higher circulating 17b-estradiol (E2) levels and increased risk of PAH in patients with cirrhosis ^36^. Inhibition of aromatase via anastrozole or metformin therapy also reduced PH and right ventricular hypertrophy in *in vivo* models of PH ^37, 38^. These observations have culminated in a randomised clinical trial in 18 patients, where anastrozole (1 mg/day) significantly reduced serum E2 levels and increased 6MWD compared with placebo ^39^, and a further phase 2 study is in progress (ClinicalTrials.gov Identifier: NCT03229499).

### Inflammation

Histological studies of PAH lung support the case for inflammation as a pathological driver of PH ^40^. A clinical trial of the anti-CD20 monoclonal antibody rituximab in PAH associated with connective tissue disease is ongoing (ClinicalTrials.gov Identifier: NCT01086540). Pre-clinical data supporting a role for interleukin-6 (IL-6) ^41^ underpins a trial of tocilizumab in PAH, which has just been completed (ClinicalTrials.gov Identifier: NCT02676947). Autoantibodies have been detected in IPAH that can contribute to worsening of the disease, and the effect of immunoadsorption as an add-on to optimised medical treatment has been explored with some haemodynamic improvement (ClinicalTrials.gov Identifier: NCT01613287). The main concern with immunomodulation is the risk of infection.

### Oxidative stress

Attempts to reduce oxidative stress in PAH have included inhibition of apoptosis signal-regulating kinase 1 (ASK1) and treatment with bardoxolone methyl. Pharmacological inhibition of ASK1 has demonstrated efficacy in a number of pre-clinical PAH models ^42^, but a phase 2 clinical trial (ClinicalTrials.gov Identifier: NCT02234141) failed to show clinical benefit.

Bardoxolone methyl is an orally available semi-synthetic triterpenoid that induces the nuclear factor erythroid 2-related factor 2 (Nrf2), a transcription factor that regulates antioxidant proteins, and suppresses activation of the pro-inflammatory factor nuclear factor kappa-light-chain-enhancer of activated B cells (NF-κB). An initial report from a phase 2 study reported some signals of efficacy, and a phase 3 study is in progress (ClinicalTrials.gov Identifiers: NCT02036970 and NCT03068130). Elamipretide, a small mitochondrially targeted tetrapeptide (D-Arg-dimethylTyr-Lys-Phe-NH2) that is currently in development as a treatment for mitochondrial disease (ClinicalTrials.gov Identifier: NCT02805790) and that appears to reduce the production of toxic reactive oxygen species (ROS) and stabilise cardiolipin, is also of interest in PH ^43^.

### Hypoxic stress and iron homeostasis

Hypoxia-inducible factor (HIF) is upregulated in remodelled pulmonary vessels. Selective deletion of either HIF1α or HIF2 offers protection against hypoxia-induced PH in mice ^44, 45^. Mutations that lead to dysfunctional von Hippel–Lindau (VHL) protein lead to PH in the context of patients with Chuvash polycythemia ^46^. Iron deficiency in the absence of anaemia is common in PAH and is associated with reduced survival ^47^. The cause is unclear. It is not explained by inflammation. Oral iron is poorly absorbed by patients with PAH. Two open-label studies of intravenous iron replacement in PAH have reported an improvement in measures of exercise capacity ^48, 49^. A randomised double-blind study is near completion (ClinicalTrials.gov Identifier: NCT01447628).

### Serotonin

The 5-HT1B receptor is highly expressed in human pulmonary arteries, has increased expression in patients with PAH, and mediates serotonin-induced vasoconstriction and remodelling ^50^. The 5-HT2A receptor mediates these effects systemically and so the 5-HT1B effects are pulmonary specific. Both the 5-HT1B receptor and serotonin transporter (SERT) are important in Nox1-derived ROS production and in serotonin-mediated vascular effects in PAH. But, so far, clinical studies evaluating pharmacological manipulation of serotonin activity in PAH have been disappointing. Current interest resides with inhibition of tryptophan hydroxylase 1 (TPH1), the rate-limiting enzyme in serotonin biosynthesis. KAR5585, a prodrug of KAR5417, is a functionally selective inhibitor of TPH1. Dose-dependent inhibition of serum serotonin and its plasma and urinary breakdown product 5-hydroxyindoleacetic acid (5-HIAA) have been demonstrated in healthy volunteers (ClinicalTrials.gov Identifier: NCT02746237). In pre-clinical PAH models, KAR5585 decreased serum, gut, and lung levels of serotonin and 5-HIAA in a dose-dependent manner and significantly reduced pulmonary arterial pressure and pulmonary vessel wall thickness and occlusion ^51^.

### Humoral modulation

Pre-clinical data ^52^ and an early clinical study suggest that vasoactive intestinal polypeptide (VIP) may have a beneficial effect in PAH ^53^. This was not supported by a study of VIP administration by inhalation ^54^, but further studies addressing the formulation and bioavailability of VIP are recruiting (ClinicalTrials.gov Identifier: NCT03315507).

Activation of the sympathetic and renin–angiotensin systems in PAH is well recognised. Although benefit from beta-blockers and inhibition of angiotensin-converting enzyme (ACE) and angiotensin II has been documented in the systemic circulation, this cannot be extrapolated to the pulmonary circulation. The debate about inhibition of sympathetic activity in PAH has been around whether it has a pathological as opposed to a compensatory role and whether interference is safe. Observations from small randomised, prospective clinical trials including bisoprolol and carvedilol have reported opposing results concerning safety ^55, 56^. Therefore, further efforts are still required to clarify the optimal timing, duration, and dosing of beta-blocker therapy in patients with PAH.

ACE2 is a homologue of ACE that is insensitive to ACE inhibitors. It converts angiotensin I and angiotensin II to angiotensin-(1–7), angiotensin-(1–9), and angiotensin-(1–5). These peptides have vasculo- and cardio-protective properties. Decreased ACE2 levels and ACE2 autoantibodies have been detected in serum from patients with PAH and have been implicated in contributing to the pathophysiology of PAH ^57^. Studies of the effect of a purified intravenous formulation of soluble recombinant human ACE 2 (rhACE2; GSK2586881) are under way (ClinicalTrials.gov Identifier: NCT03177603), but a recent open-label pilot study of five PAH patients (ClinicalTrials.gov Identifier: NCT01884051) found that a single infusion of rhACE2 was well-tolerated and was associated with improved pulmonary haemodynamics and reduced markers of oxidant and inflammatory stress ^58^.

Interest in the fibrogenic properties of aldosterone has led to studies of mineralocorticoid receptor antagonism with spironolactone or eplerenone in animal models, which provide evidence of efficacy. Retrospective analysis of data on the addition of spironolactone to targeted PAH treatment with ambrisentan from ARIES 1 and ARIES 2 studies suggests some clinical benefit. Two prospective studies are examining the effect of chronic spironolactone on markers of fibrosis (ClinicalTrials.gov Identifier: NCT01468571) and exercise capacity (ClinicalTrials.gov Identifier: NCT01712620).

### Epigenetic

Histone deacetylase (HDAC) inhibitors have been reported to produce both benefit ^59, 60^ and harm ^61^ in pre-clinical models of PAH. Of particular concern is cardiotoxicity. There are four classes of HDACs and a number of subtypes. Identifying the HDAC subtype that is relevant and a specific inhibitor is key to unlocking the therapeutic potential of this approach. Indeed, recent pre-clinical studies suggest that HDAC6 is implicated in the development of PAH and selective inhibition represents a new promising target to improve PAH ^62^. MicroRNAs (miRNAs) have also been shown to reverse as well as prevent PH in animal models ^63^. As with HDAC inhibitors, the challenge in the first instance is to identify the most relevant miRNA in PAH pathobiology and attention has focused on the miR-143/145 cluster, for example 64, 65.

### DNA damage

Dysregulation of DNA damage-and-repair mechanisms has been identified as a trigger for disease progression in PAH ^66^, and inhibition of poly(ADP-ribose) polymerase (PARP) reverses PAH in several animal models ^67^. A safety study to repurpose olaparib, an orally available PARP inhibitor approved for the treatment of BRCA-related breast cancer, for PAH has been proposed (ClinicalTrials.gov Identifier: NCT03251872).

## Challenges

With these opportunities come challenges. The animal models are not high-fidelity reproductions of the human condition and have a poor track record for predicting efficacy. The clinical trial design template used for the currently approved drugs, which act by reducing vascular tone, is not suitable for the development of drugs that target vascular remodelling. Clinical trials have to compete for a relatively small pool of patients.

There is considerable room to improve how we select and prioritise new candidate drugs for evaluation, how we identify suitable patients for each study, how we use creative trial designs for go/no-go decisions, and how we learn from failure. Targets suggested by genetic studies understandably hold pride of place in prioritising candidates to take forward. A variant that is associated with PAH through a biologically plausible pathway (that is, supported by endophenotype data) reduces the risk of failure and provides a biomarker for selecting suitable patients for the clinical study. But we can also learn from studies where human exposure has already taken place. Analysing samples and data from patients according to response can suggest strategies for enrichment (using genetic, proteomic, metabolomic, or other clinical markers) in follow-on studies with patients more likely to respond.
